# CircRNA Cdyl promotes the proliferation and differentiation of neural stem cells via regulating miR-544-3p/Nr3c1 axis

**DOI:** 10.1016/j.isci.2026.114716

**Published:** 2026-01-15

**Authors:** Wen Li, Yujian Lin, Jingwen Wang, Zuotian Zhang, Jingjing Zhang, Yuzheng Zhang, Yuxuan Ge, Tiankun Yao, Xiang Cheng, Weiwei Chen, Min Xu, Xinhua Zhang

**Affiliations:** 1 Department of Human Anatomy, Co-Innovation Center of Neuroregeneration, Nantong University, No.19 Qixiu Road, Nantong, Jiangsu 226001, P.R. China; 2 Clinical Trial Center, Yancheng Third People’s Hospital, The Sixth Affiliated Hospital of Nantong University, Yancheng 224002, China

**Keywords:** Neuroscience, Molecular neuroscience, Developmental neuroscience, Cellular neuroscience

## Abstract

Emerging evidence suggests that numerous circular RNAs (circRNAs) are abnormally expressed during neural stem cell (NSC) differentiation, which may play an important role in the fate of NSCs. Here, we explored the potential mechanism of circRNA Cdyl (circCdyl) in NSC proliferation and differentiation. SD rats underwent fimbria-fornix (FF) transection, while primary NSCs were induced to proliferate and differentiate. CCK8, qRT-PCR, Western blot, flow cytometry, and immunofluorescence assay to confirm the function of circCdyl *in vitro* and *in vivo*. The results showed that circCdyl was elevated during NSC proliferation and differentiation, and in the hippocampus after FF transection. Besides, circCdyl overexpression promoted the proliferation and neuronal differentiation of hippocampal NSCs through miR-544-3p/subfamily 3 group C member 1 (Nr3c1) axis. *In vivo* experiments revealed that the overexpression of circCdyl promoted hippocampal neurogenesis and improved cognitive dysfunction after FF transection, which provides a potential therapeutic target for cognitive dysfunction.

## Highlights

• circCdyl was elevated in the hippocampus after FF transection
• circCdyl promoted the proliferation of NSCs through miR-544-3p/Nr3c1 axis
• circCdyl promoted the neuronal differentiation of NSCs through the miR-544-3p/Nr3c1 axis
• *In vivo* circCdyl promoted hippocampal neurogenesis and improved cognitive dysfunction

## Graphical abstract

**Figure:**
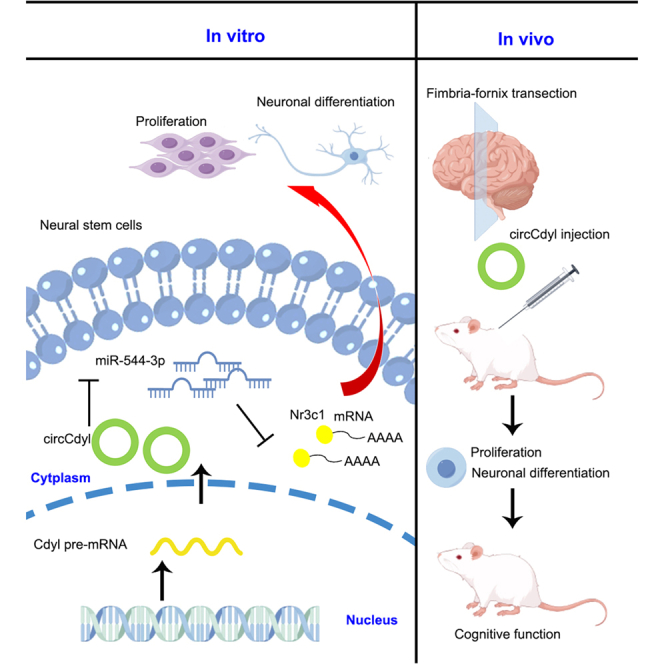

## Introduction

A wide range of cognitive dysfunction, including progressive memory loss, has been attributed to the disrupted hippocampal performance.^1^ In the adult brain, the dentate gyrus (DG) of the hippocampus is a specialized niche, which is involved in the generation of newborn neurons.^2^ Recent studies found that as Alzheimer's disease (AD) advanced, the number and maturity of newborn neurons in the hippocampus progressively declined,^3^^,^^4^ suggesting that the decline might exacerbate cognitive deficits. Within the past decade, NSCs have been the optimal cells for repairing damage in the central nervous system due to their capacity for self-renewal and, most importantly, their ability to differentiate into mature, functional neurons.^5^ NSCs have been investigated as a potential therapeutic approach, including transplanting exogenous NSCs and boosting the self-repair of endogenous NSCs.^6^ Understanding the potential of NSCs and their niche components is essential for the development of therapies against neurological disorders.

Circular RNAs (circRNAs) are a unique class of endogenous non-coding RNA formed through back-splicing of linear RNA.^7^ Increasing studies have reported that circRNAs play crucial roles in the regulation mechanism of various diseases, such as tumors, neurological diseases, and inflammation.^8^

Research indicates that circRNAs are expressed across all rat tissues and enriched in the brain, where their levels were regulated in an organ-, development and gender-specific manner.^9^ Numerous brain-enriched circRNAs have been implicated in critical processes during brain development, including neurotransmitter function, neuronal maturation, and synaptic activity.^9^^,^^10^ For example, a circRNA derived from the Homer Scaffolding Protein 1 pre-RNA (circHomer1) has been shown to putatively facilitate synaptic plasticity during neuronal plasticity and development.^11^ Furthermore, circTLK1 is generated from the TLK1 gene, which is thought to aggravate neuronal injury and neurological deficits after ischemic stroke.^12^

Recently, the competitive endogenous RNA (ceRNA) regulatory model has described circRNA as a sponge for miRNA, thereby indirectly regulating downstream target genes.^13^ A lot of studies indicate that the crosstalk among circRNAs, miRNAs, and mRNAs plays a crucial role in modulating both physiological and pathological processes in the brain. For example, circRNA CDR1as, also known as ciRS-7, a gigantic molecule, functions as a miR-7 sponge to regulate UBE2A, which is involved in the accumulation of Aβ and the formation of senile plaque deposits.^14^ Similarly, circRNA DLGAP4 functions as a sponge of miR-134-5p, influencing CREB expression and exerting neuroprotective effects in Parkinson’s disease.^15^ Elucidating the regulatory mechanisms underlying circRNA-miRNA-mRNA networks will be critical for identifying potential therapeutic targets for cognitive disorders of the brain.

Previous studies have demonstrated that exosomes derived from the denervated hippocampus could promote the differentiation of NSCs into neurons. Moreover, the differentially expressed circRNAs in exosomes were detected by RNA-seq, among which circCdyl was found to be the most significantly upregulated.^16^^,^^17^ CircCdyl is spliced from exons of Cdyl on the reverse strand of chromosome17. To date, evidence on the biological functions of circCdyl in NSCs remains limited. In this study, we verified that circCdyl, as a competing endogenous RNA (ceRNA) for miR-544-3p, upregulated the expression of Nr3c1, which contributed to NSC proliferation and differentiation into neurons. In addition, circCdyl could improve cognitive function after fimbria-fornix (FF) transection and promote hippocampal neurogenesis.

## Results

### Identification and characterization of circular RNAs Cdyl

**Figure 1:**
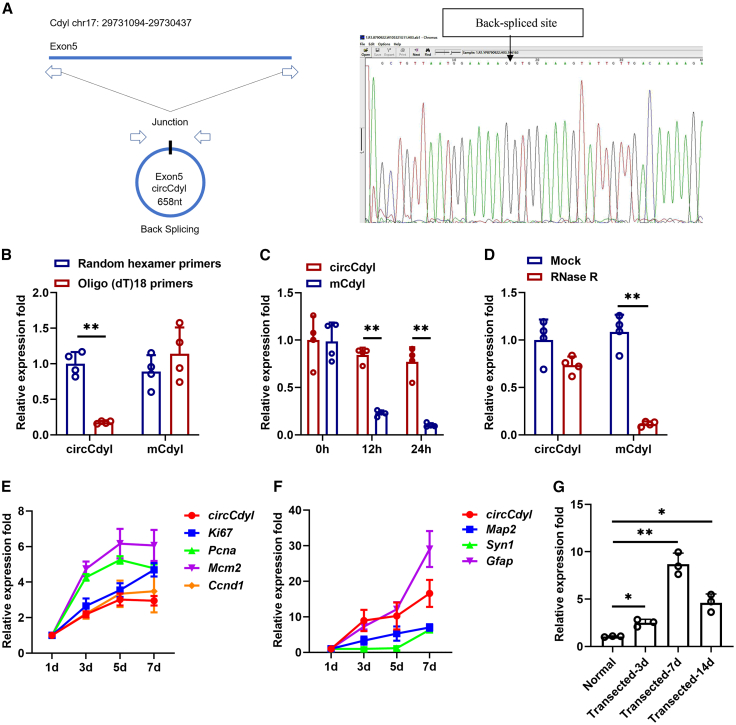
Identification and characterization of circCdyl (A) Schematic diagram of the generation of circCdyl (left). To validate the existence of circCdyl, primers were designed on the spliced junction, followed by Sanger sequencing (right). (B) Random hexamer or oligo(dT)18 primers were used in the reverse transcription. The RNA levels were analyzed by qRT-PCR. (C) qRT-PCR was conducted to determine the abundances of circCdyl and Cdyl in NSCs after treatment for 12 h and 24 h with actinomycin D. (D) The abundance of circCdyl and Cdyl mRNA in NSCs treated with RNase R was detected by qRT-PCR. (E and F) qRT-PCR was performed to detect the existence of circCdyl in proliferative and differentiated states of NSCs at the indicated time points. (G) qRT-PCR was performed to detect the existence of circCdyl in the hippocampus after FF. n = 3–4 in each group. All data are presented as mean ± SD and analyzed by paired Student’s *t* test. ∗*p* < 0.05, ∗∗*p* < 0.01, ∗∗∗*p* < 0.001, values significantly different from corresponding control group.

circCdyl is derived from exon5 of the Cdyl gene. NSCs were isolated from the hippocampus of E15 SD rats as described previously.^18^ To validate the existence of circCdyl, divergent primers were designed to amplify the circCdy in NSCs, confirmed by Sanger sequencing (Figure 1A). To confirm the circular characteristics, random hexamer or oligo (dT)18 primers were used. Compared with random hexamer primers, the relative expression of circCdyl was barely detected when the primers were replaced by oligo (dT) 18 (Figure 1B). Actinomycin D was used to evaluate the stability of circCdyl. The results showed that circCdyl was more stable than linear Cdyl mRNA (Figure 1C). RNase R digestion confirmed that circCdyl was resistant to digestion with RNase R exonuclease (Figure 1D). Subsequently, qRT-PCR was used to detect the circCdyl expression of NSCs in their proliferative (proliferation-related markers: Ki67, Pcna, Mcm2, and Ccnd1) and differentiated states (neuron-related markers: Map2 and Tuj1; astrocyte-related markers: Gfap) at different time points. The results showed that circCdyl expression gradually increased during NSC proliferation and differentiation (Figures 1E and 1F). Similarly, we found circCdyl gradually increased and reached a peak on the 7th day in the hippocampus after FF transection (Figure 1G).

### Circular RNAs Cdyl promotes neural stem cell proliferation and differentiation into neurons

**Figure 2:**
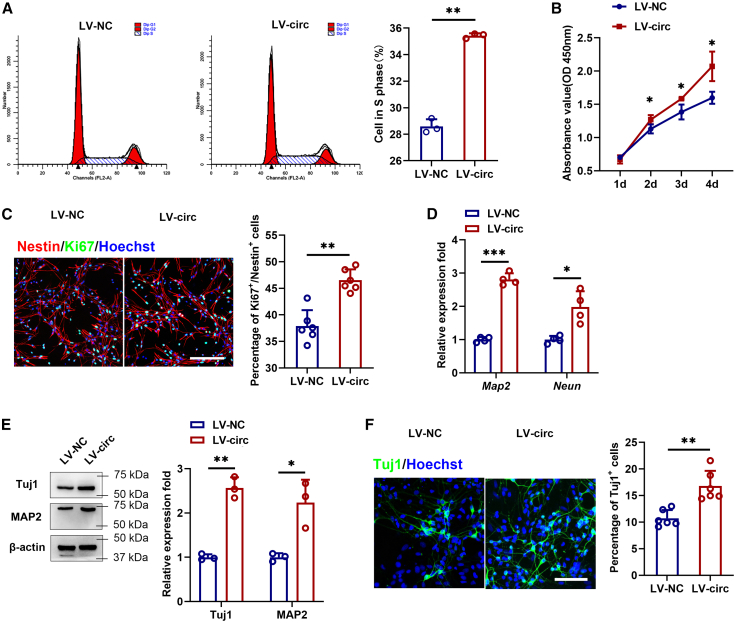
Functions of circCdyl in NSCs (A) Cell cycle distribution was analyzed by flow cytometry. (B) CCK8 assay of NSCs was analyzed at the indicated days. (C) Immunofluorescence assay was performed to evaluate NSC proliferation (magnification, ×20, Scale bars, 200 μm). (D and E) The expression of neuronal marker was measured by qRT-PCR and Western blot. (F) Cell differentiation was also detected by immunofluorescence (magnification, ×20, Scale bars, 100 μm). (LV-NC) NSCs infected with control lentivirus; (LV-circ) NSCs infected with circCdyl overexpression of lentivirus. n = 3–6 in each group. All data are presented as mean ± SD and analyzed by paired Student’s *t* test. ∗*p* < 0.05, ∗∗*p* < 0.01, ∗∗∗*p* < 0.001, values significantly different from corresponding control group.

In our study, circCdyl was overexpressed in NSCs to investigate its role in proliferation and differentiation. The results demonstrated that following the overexpression of circCdyl, its expression was significantly upregulated, whereas the expression level of Cdyl remained unchanged. (Figure S1A). Flow cytometric analysis revealed a significant increase in the proportion of cells in the S phase following circCdyl overexpression (Figure 2A). CCK8 assays further demonstrated that circCdyl overexpression significantly enhanced the proliferation ability of NSCs (Figure 2B). Consistently, immunofluorescence assays showed that the percentage of Ki67-positive cells was increased among Nestin-positive NSCs compared with control (Figure 2C). NSCs transduced with circCdyl overexpression lentivirus or control lentivirus were induced to differentiate for 7 days, followed by the assessment of neuronal markers Tuj1, MAP2, and NeuN. The results showed that the expression of neuronal markers increased significantly after circCdyl overexpression (Figures 2D and 2E). Immunofluorescence assay revealed that circCdyl promoted cell differentiation into neurons (Figure 2F). In addition, astrocytic marker expression was evaluated using qRT-PCR and flow cytometry, which revealed no significant effect of circCdyl on astrocyte differentiation (Figure S2).

### Circular RNAs Cdyl promotes the recovery of cognitive function after fimbria-fornix transection

**Figure 3:**
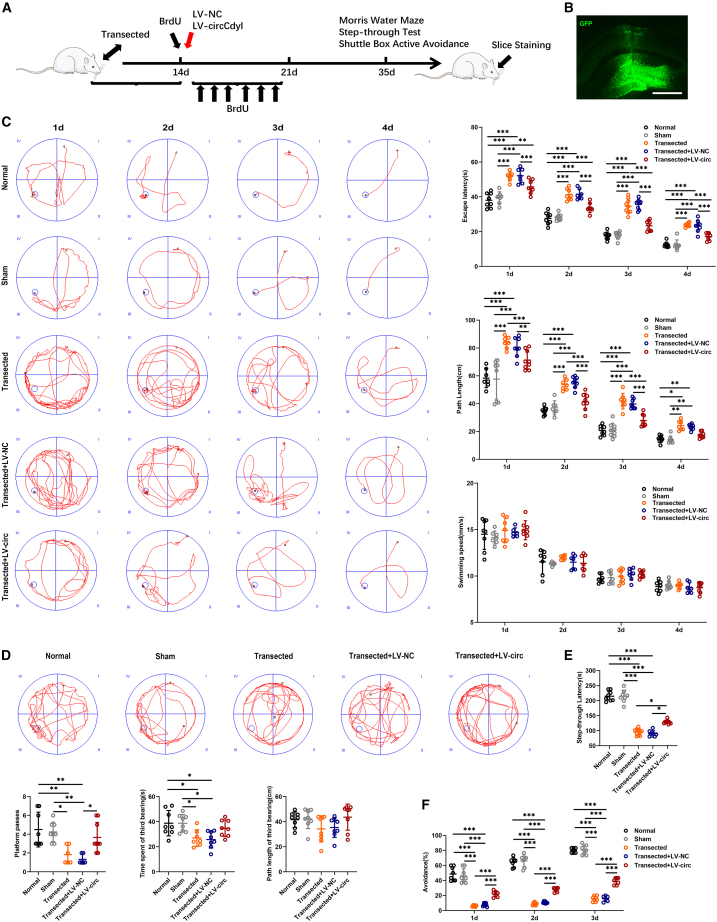
Upregulation of circCdyl improves cognitive function *in vivo* (A) The schedule of *in vivo* experiments. (B) lentivirus infection in the hippocampus. (C) The Morris water maze test was performed to analyze learning performance. Latency to the platform and path length, and speed were measured. (D) Times traveling across the platform, time spent in quadrant 3, and path length in quadrant 3 were measured. (E and F) The passive and active avoidance behavior of rats by shuttle box test. Step-through latency and the rate of active avoidance were measured. (Normal) received no any treatment; (Sham) received only craniotomy, but no FF transection; (Transected) received only FF transection; (Transected+LV-NC) received FF transection and control lentivirus; (Transected+LV-circ) received FF transection and circCdyl overexpression lentivirus. *n* = 8 in each group. All data are presented as mean ± SD and analyzed by one-way ANOVA followed by Tukey’s multiple comparison test. ∗*p* < 0.05, ∗∗*p* < 0.01, ∗∗∗*p* < 0.001, values significantly different from corresponding control group.

To examine the effects of circRNA on cognitive function, we microinjected lentivirus into the hippocampus of SD rats. As shown in Figure 3A, the timeline of the experimental procedure in our research. GFP fluorescence in the hippocampus confirmed successful lentiviral transduction of target tissues (Figure 3B), and qRT-PCR confirmed overexpression of circCdyl in the hippocampus (Figure S1E). In the Morris water maze, circCdyl improved spatial learning as indicated by reduced escape latency, path length, and a greater number of platform crossings (Figures 3C and 3D). In the retention phase, the latency of the circCdyl overexpression group was significantly higher than that of the control group (Figure 3E). In terms of the number of active avoidance, SD rats in the circCdyl overexpression group performed more times than the control group, and increased in a time-dependent manner (Figure 3F).

### Circular RNAs Cdyl promotes hippocampal neurogenesis

**Figure 4:**
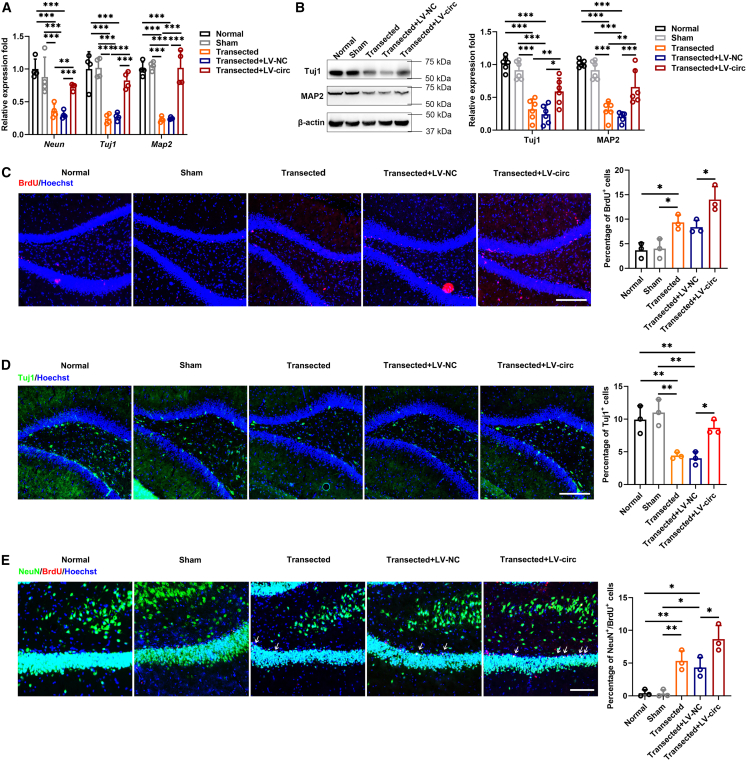
Upregulation of circCdyl promotes hippocampal neurogenesis *in vivo* (A and B) The expression of neuronal marker was measured by qRT-PCR and Western blot. (C) BrdU^+^ cells in the DG of hippocampal (magnification, ×20, Scale bars, 200 μm). (D) Tuj1^+^ cells in the DG of hippocampal (magnification, ×20, Scale bars, 200 μm). (E) NeuN^+^/BrdU^+^ cells in the DG of hippocampal (magnification, ×40, Scale bars, 100 μm). (Normal) received no any treatment; (Sham) received only craniotomy, but no FF transection; (Transected) received only FF transection; (Transected+LV-NC) received FF transection and control lentivirus; (Transected+LV-circ) received FF transection and circCdyl overexpression lentivirus. n = 3–6 in each group. All data are presented as mean ± SD and analyzed by one-way ANOVA followed by Tukey’s multiple comparison test. ∗*p* < 0.05, ∗∗*p* < 0.01, ∗∗∗*p* < 0.001, values significantly different from corresponding control group.

By qRT-PCR and Western blot, we detected the expression of neuronal markers in the hippocampus after treatment, and the results showed that Tuj1, MAP2, and NeuN were significantly up-regulated (Figures 4A and 4B). Next, the number of BrdU, Tuj1, and NeuN was evaluated with immunofluorescence. The results showed that BrdU^+^ cells, Tuj1^+^ cells, and NeuN^+^/BrdU^+^ cells were significantly increased in the circCdyl overexpression group compared with the control group (Figures 4C–4E).

### Circular RNAs Cdyl directly binds to miR-544-3p and suppresses miR-544-3p activity

**Figure 5:**
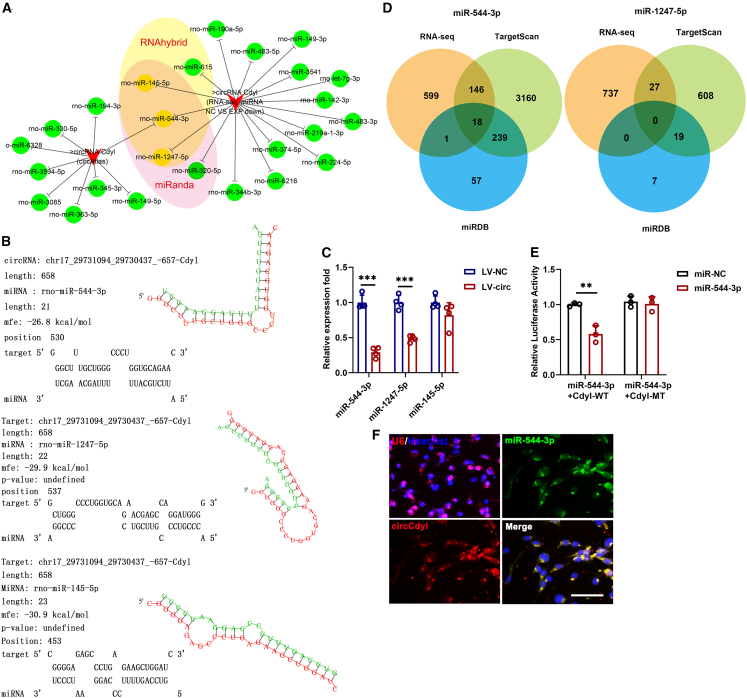
circCdyl targets miR-544-3p (A) Target genes were analyzed using three publicly available algorithms (circAtlas, RNAhybrid and miRanda) and RNA-seq. (B) Bioinformatics predicted the binding sites of target genes with circCdyl. (C) qRT-PCR was conducted to determine the abundances of miR-145-5p, miR-544-3p, miR-1247-5p in NSCs after circCdyl overexpression. (LV-NC) NSCs infected with control lentivirus; (LV-circ) NSCs infected with circCdyl overexpression of lentivirus. (D) Target genes of miR-544-3p and miR-1247-5p were analyzed using two publicly available algorithms (TargetScan and miRDB) and RNA-seq. (E) Luciferase activity of circCdyl wild types and mutants in 293T cells transfected with miR-544-3p mimic and negative control mimic. (F) FISH for circCdyl and miR-544-3p in NSCs (magnification, ×40, Scale bars, 100 μm). n = 3–4 in each group. All data are presented as mean ± SD and analyzed by paired Student’s *t* test. ∗∗*p* < 0.01, ∗∗∗*p* < 0.001, values significantly different from the corresponding control group.

Using three publicly available algorithms (circAtlas, RNAhybrid, and miRanda) combined with RNA-seq analysis, we identified miR-145-5p, miR-544-3p, and miR-1247-5p as potential targets of circCdyl (Figure 5A), with predicted binding sites shown in Figure 5B circCdyl overexpression significantly reduced the relative abundance of miR-544-3p and miR-1247-5p (Figure 5C). Furthermore, using two prediction algorithms (TargetScan and miRDB) and RNA-seq data, we identified 18 potential target genes for miR-544-3p, but none for miR-1247-5p (Figure 5D). Luciferase reporter assays demonstrated that miR-544-3p suppressed the luciferase activity of wild-type circCdyl, but not that of the mutant construct (Figure 5E). Fluorescence *in situ* hybridization (FISH) revealed the colocalization of circCdyl and miR-544-3p in NSCs (Figure 5F).

### Circular RNAs Cdyl, coordinated with miR-544-3p, regulates neural stem cell proliferation and differentiation

**Figure 6:**
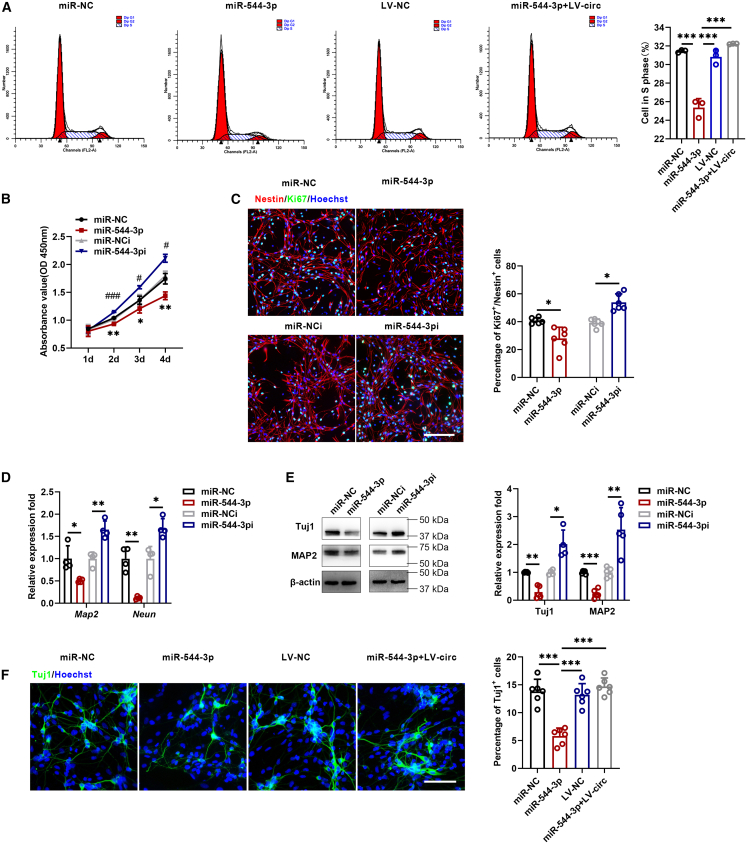
circCdyl coordinated with miR-544-3p, regulates NSC proliferation and differentiation (A) Cell cycle distribution was analyzed by flow cytometry. (B) CCK8 assay of NSCs was analyzed at the indicated days. (C) Immunofluorescence assay was performed to evaluate NSC proliferation (magnification, ×20, Scale bars, 200 μm). (D and E) The expression of neuronal marker was measured by qRT-PCR and Western blot. (F) Cell differentiation was also detected by immunofluorescence (magnification, ×20, Scale bars, 100 μm). (miR-NC) NSCs transfected with control miRNA mimic; (miR-544-3p) NSCs transfected with miR-544-3p mimic; (miR-NCi) NSCs transfected with control miRNA inhibitor; (miR-544-3pi) NSCs transfected with miR-544-3p inhibitor; (LV-NC) NSCs infected with control lentivirus; (miR-544-3p+LV-circ) NSCs transfected with miR-544-3p mimic and circCdyl overexpression of lentivirus. n = 3–6 in each group. All data are presented as mean ± SD and analyzed by one-way ANOVA followed by Tukey’s multiple comparison test or paired Student’s *t* test. ∗*p* < 0.05, ∗∗*p* < 0.01, ∗∗∗*p* < 0.001, values significantly different from corresponding control group.

In this study, miR-544-3p was overexpressed or inhibited to investigate its functional role in NSCs (Figure S1B). Flow cytometry, CCK8 assays, and immunofluorescence staining were performed to evaluate the effects of miR-544-3p on NSC proliferation. The results showed that miR-544-3p overexpression suppressed NSC proliferation, whereas knockdown of miR-544-3p promoted cell proliferation. Furthermore, circCdyl overexpression rescued the proliferative capacity in these cells (Figures 6A–6C). We next examined whether miR-544-3p regulates neuronal differentiation. qRT-PCR, Western blot, and immunofluorescence analyses demonstrated that the inhibition of miR-544-3p significantly increased the expression of neuronal markers, while the overexpression of miR-544-3p reduced their expression. Moreover, the inhibitory effect of miR-544-3p on neuronal differentiation was reversed by circCdyl overexpression (Figures 6D–6F).

### miR-544-3p targets Nr3c1

**Figure 7:**
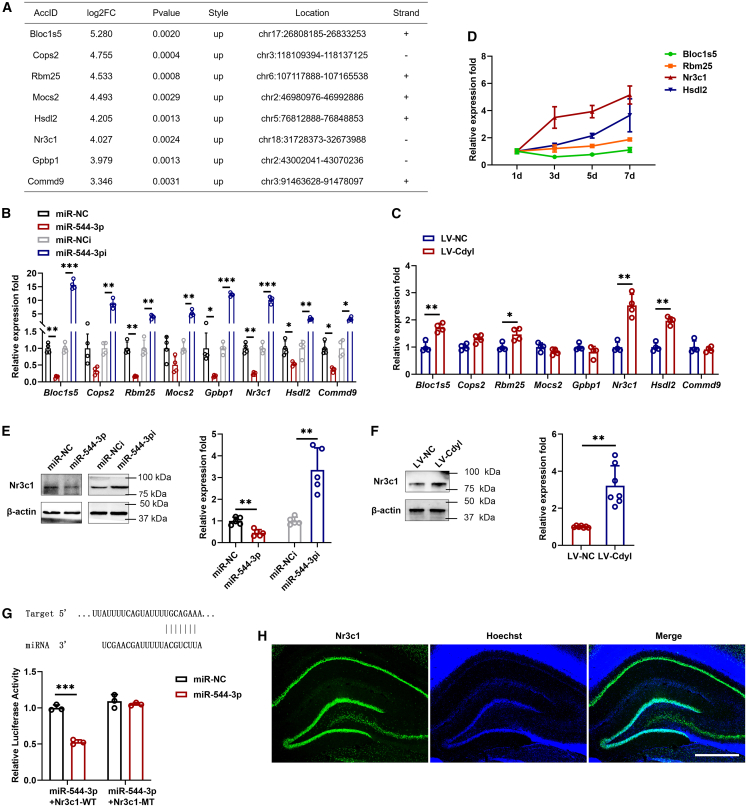
Nr3c1 is a direct target of miR-544-3p (A) Table listing the top 8 candidates including the AccID, Log2FC, PValue, Style, Location and Strand. (B) qRT-PCR was conducted to determine the abundances of potential target genes in NSCs transfected with miR-544-3p. (miR-NC) NSCs transfected with control miRNA mimic; (miR-544-3p) NSCs transfected with miR-544-3p mimic; (miR-NCi) NSCs transfected with control miRNA inhibitor; (miR-544-3pi) NSCs transfected with miR-544-3p inhibitor. (C) qRT-PCR was conducted to determine the abundances of potential target genes in NSCs after circCdyl overexpression. (LV-NC) NSCs infected with control lentivirus; (LV-circ) NSCs infected with circCdyl overexpression of lentivirus. (D) qRT-PCR was performed to detect the existence of potential target genes in NSCs at the indicated time points. (E and F) Western blot was conducted to determine the abundances of Nr3c1 in NSCs transfected with miR-544-3p or circCdyl. (G) Luciferase activity of Nr3c1 wild types and mutants in 293T cells transfected with miR-544-3p mimic and negative control mimic. (H) Expression of Nr3c1 in the hippocampus of DG was also detected by immunofluorescence (magnification, ×10, Scale bars, 400 μm). n = 3–7 in each group. All data are presented as mean ± SD and analyzed by paired Student’s *t* test. ∗*p* < 0.05, ∗∗*p* < 0.01, ∗∗∗*p* < 0.001, values significantly different from corresponding control group.

Using two publicly available algorithms (TargetScan and miRDB) combined with RNA-seq analysis, we identified 18 potential target genes of miR-544-3p (Figure 5D). Among these, genes showing at least a 3-fold enrichment in RNA-seq were selected for further analysis. The top 8 most highly enriched targets were validated by qRT-PCR (Figure 7A). Given that the overexpression of miR-544-3p was unable to alter the expression abundance of Cops2 and Mocs2, these genes were not analyzed further (Figure 7B). In addition, there were no significant differences in the expression levels of Gpbp1 and Commd9 after circCdyl overexpression (Figure 7C). We initially focused on four target genes, Blocls5, Rbm25, Nr3c1, and Hsdl2. qRT-PCR results revealed that Nr3c1 expression was significantly upregulated in a time-dependent manner (Figure 7D). Western blot also confirmed that the expression abundance of Nr3c1 was significantly changed after treatment with miR-544-3p and circCdyl (Figures 7E and 7F). Next, wild and mutant dual-luciferase reporter plasmids containing Nr3c1 3′-UTRs were constructed. We found that the overexpression of miR-544-3p evidently reduced luciferase activity (Figure 7G). Furthermore, immunofluorescence showed that Nr3c1 was enriched in the granular layer of the DG of the hippocampus (Figure 7H). After FF, the expression of Nr3c1 gradually increased and reached a peak on the 7th day (Figure S3A). Western blot showed that compared with the normal group, the expression of Nr3c1 was significantly upregulated in the Transected-7d group (Figure S3B).

### Circular RNAs Cdyl regulates Nr3c1 by sponging miR-544-3p to promote neural stem cell proliferation and differentiation

**Figure 8:**
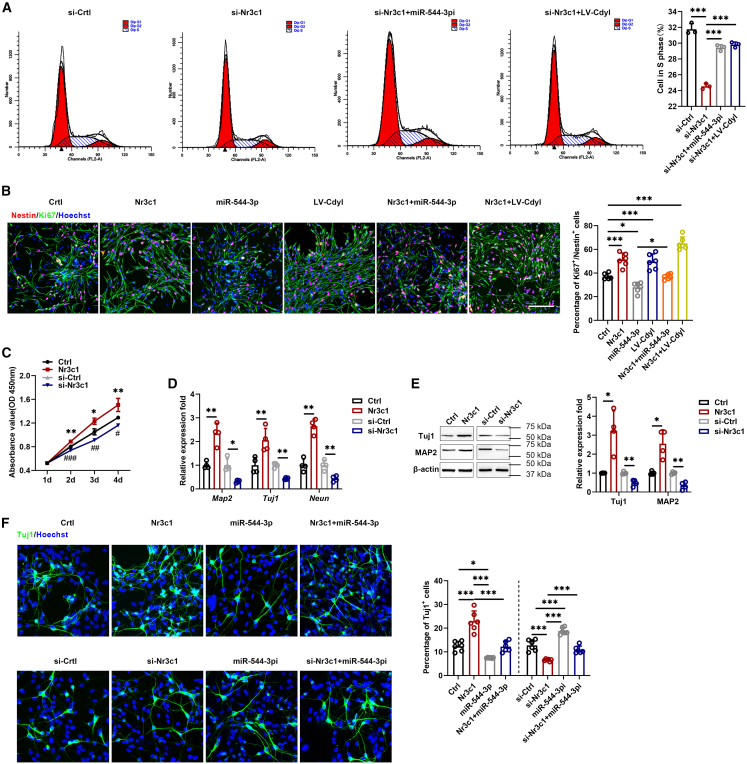
CircCdyl regulates Nr3c1 by sponging miR-544-3p to promote NSC proliferation and differentiation (A) Cell cycle distribution was analyzed by flow cytometry. (B) Immunofluorescence assay was performed to evaluate NSC proliferation (magnification, ×20, Scale bars, 200 μm). (C) CCK8 assay of NSCs was analyzed at the indicated days. (D and E) The expression of neuronal marker was measured by qRT-PCR and Western blot. (F) Cell differentiation was also detected by immunofluorescence (magnification, ×20, Scale bars, 100 μm). (Ctrl) NSCs transfected with control pcDNA; (Nr3c1) NSCs transfected with pcDNA Nr3c1; (si-Ctrl) NSCs transfected with control siRNA; (si-Nr3c1) NSCs transfected with Nr3c1 siRNA; (miR-544-3p) NSCs transfected with miR-544-3p mimic; (miR-544-3pi) NSCs transfected with miR-544-3p inhibitor; (LV-circ) NSCs infected with circCdyl overexpression of lentivirus; (Nr3c1+miR-544-3p) NSCs transfected with pcDNA Nr3c1 and miR-544-3p mimic; (si-Nr3c1+miR-544-3pi) NSCs transfected with Nr3c1 siRNA and miR-544-3p inhibitor; (Nr3c1+LV-circ) NSCs transfected with pcDNA Nr3c1 and circCdyl overexpression of lentivirus. (si-Nr3c1+LV-circ) NSCs transfected with Nr3c1 siRNA and circCdyl overexpression of lentivirus. n = 3–6 in each group. All data are presented as mean ± SD and analyzed by one-way ANOVA followed by Tukey’s multiple comparison test or paired Student’s *t* test. ∗*p* < 0.05, ∗∗*p* < 0.01, ∗∗∗*p* < 0.001, values significantly different from corresponding control group.

In our study, Nr3c1 was overexpressed or inhibited to examine the function of Nr3c1 in proliferation and differentiation (Figure 1, Figure 2, Figure 3, Figure 4, Figure 5, Figure 6, Figure 7, Figure 8C and S1D). Flow cytometry, CCK8, and immunofluorescence assay revealed that Nr3c1 overexpression promoted the proliferation of NSCs. The proliferative effect of Nr3c1 was inhibited by miR-544-3p. Conversely, knockdown of Nr3c1 inhibited cell proliferation. In addition, the proliferation abilities were cooperatively increased after circCdyl overexpression (Figures 8A–8C). qRT-PCR, Western blot, and immunofluorescence analysis showed that Nr3c1 overexpression significantly increased expression levels of neuronal markers, whereas the expression was decreased after Nr3c1 silencing. Besides, the promoting effect of Nr3c1 on the differentiation of NSCs into neurons was also inhibited after miR-544-3p overexpression (Figures 8D–8F).

## Discussion

Adult stem cells are the essential source of building blocks for tissue homeostasis, growth, and regeneration.^19^ The subgranular zone (SGZ) of the DG in the hippocampus is the neurogenic niche in the brain, where resident adult NSCs can generate new neurons and glial cells.^20^ Most adult NSCs are maintained in a reversible state of cell-cycle arrest. Upon activation, they proliferate and differentiate into immature neurons, which subsequently mature into DG neurons and integrate into existing neuronal circuits.^21^ The hippocampus plays an important role in spatial and episodic memory, which is highly vulnerable to damage in multiple neurodegenerative diseases.^22^ If endogenous adult hippocampal neurogenesis can be facilitated to promote regeneration, then the cognitive impairment caused by hippocampal dysfunction could be ameliorated.

A previous study demonstrated that the internal microenvironment changes following FF transection lead to the activation of endogenous hippocampal NSCs, promoting their proliferation, migration, survival, and neuronal differentiation.^23^ Furthermore, we found that exosomes derived from denervated tissue, when cocultured with NSCs, enhance the differentiation of hippocampal NSCs into neurons and cholinergic neurons. High-throughput transcriptome sequencing identified upregulated circRNAs in hippocampal exosomes after FF transection,^16^ supporting a functional role for exosomes and their molecular cargo in regulating hippocampal neurogenesis. In conclusion, our findings highlight hippocampus-secreted exosomes and their circRNA cargo as key contributors to hippocampal nerve regeneration. Understanding the regulatory functions of circRNAs in hippocampal neurogenesis provides a foundation for harnessing NSCs in the treatment of central nervous system disorders.

circRNAs are covalently closed RNA molecules generated through back-splicing of precursor mRNAs.^24^ By harboring microRNA response elements (MREs), circRNAs can competitively bind to miRNAs and thereby regulate diverse biological processes.^25^ Accumulating evidence reveals that circRNAs are highly enriched and dynamically expressed in the brain.^10^ Altered expression of circRNAs in the brain is often regulated in tissue-specific and development-dependent manner.^26^ For instance, Rybak-Wolf et al.^27^ found that region-specific circRNAs are expressed in the prefrontal cortex, olfactory bulb, cerebellum, and hippocampus of the mouse brain. Using high-resolution *in situ* hybridization, You et al.^28^ visualized several synaptic function-related circRNAs, including circHomer1, circDscam, circKlhl2, circElavl3, circNlgn1, circGigyf2, circNbea, and circRmst.

Interestingly, studies have shown that circRNAs are enriched and stable in exosomes compared to their producer cells. Li et al.^29^ found that the ratio of circRNA level in exosomes was about 2-fold higher than that in producer cells. Besides, exosomal circRNAs can be regulated by relevant miRNA levels in donor cells, and this molecular cargo is subsequently transferred to recipient cells.^30^ For example, exosomal circRASSF2 acts as a miR-302b-3p sponge, significantly inhibiting cell proliferation and migration.^31^ Similarly, exosomal circNRIP1 sponges miR-149-5p to promote gastric cancer progression via the AKT1/mTOR pathway.^32^ Accumulating evidence has identified a functional role for exosomal circRNAs in physiological and pathological processes of the central nervous system. For example, Zhao et al.^33^ showed that exosomal circRNAs might be related to the growth and repair of neurons, the development of the nervous system, and the transmission of nerve signals, specifically in glutamatergic synapses and the cGMP-PKG signaling pathway. CircRNA KIAA1586 occurred most frequently in the AD-related ceRNA network, and binds competitively to three known AD-risk miRNAs.^34^ Understanding the function and characteristics of exosomal circRNAs provides a novel approach for disease diagnoses and targeted Therapies.

Based on these reports, we analyzed the expression pattern of the circCdyl in NSCs. CircCdyl is conserved in humans, and its expression has been confirmed in various diseases, such as tumor processes^35^^,^^36^ and cardiac hypertrophy.^37^ There were almost no reports on the relationship between circCdyl and NSCs. However, the host gene of circCdyl was discovered that Cdyl mutation disrupts neuronal migration and increases susceptibility to epilepsy.^38^ We have previously found that FF transection provided a proper microenvironment for the survival and neuronal differentiation of hippocampal NSCs.^23^ In this study, with the increase of NSC proliferation and differentiation, circCdyl expression was increased gradually. Similarly, we found that circCdyl significantly increased in hippocampal tissue after FF transection. It suggests that circCdyl might play key roles in neural biological processes. Then, following circCdyl overexpression, more NSCs proliferated and differentiated into neurons. Moreover, the experiments *in vivo* showed that circCdyl could significantly improve learning and memory ability and promote hippocampal neurogenesis. It provided new evidence for the role of circCdyl in the regulation of NSC proliferation and differentiation.

To further elucidate the mechanisms underlying circCdyl-mediated NSC proliferation and differentiation, we constructed networks of CircCdyl. According to the union of target prediction sites, we found that miR-544-3p and miR-1247-5p may be the target genes of circCdyl. Since miR-1247-5p had no predicted downstream target genes, it was excluded from further analysis. The direct interaction between circCdyl and miR-544-3p was clearly observed under confocal microscopy at 40× magnification. Importantly, cell phenotypes were rescued when overexpressing the CircCdyl in miR-544-3p overexpression cells, implying miR-544-3p as an essential regulator in this model.

Based on the ceRNA hypothesis, we found that Nr3c1 is a latent target of miR-544-3p. Subsequently, the dual-luciferase reporter assay verified that miR-544-3p might directly bind to the 3′-UTR of Nr3c1. We further found that Nr3c1 was enriched in the granular layer of the DG of the hippocampus. Moreover, Nr3c1 was significantly up-regulated with the increasing of NSC proliferation and in hippocampal tissue after FF transection. These discoveries suggested that Nr3c1 may play an important role in neurogenesis. Nr3c1 is a member of the nuclear receptor subfamily involved in the regulation of DNA methyltransferase. Studies have shown that Nr3c1 has been associated with early life stress and psychosocial stress reactivity.^39^ Furthermore, Nr3c1 is a neuron-specific glucocorticoid receptor that is strongly related to adult hippocampal neurogenesis and cognitive deficits.^39^ Here, we revealed that the upregulation of CircCdyl could enhance the expression of Nr3c1 as well as promote NSC proliferation and differentiation into neurons. More importantly, upregulation of CircCdyl could enhance the function of Nr3c1, whereas these impacts might be reversed by miR-544-3p mimic. These results further confirm our hypothesis that CircCdyl acts as a ceRNA for miR-544-3p to enhance Nr3c1 expression and regulate NSC proliferation and differentiation into neurons.

In summary, we identified a novel circCdyl that functions as an endogenous sponge for miR-544-3p to regulate Nr3c1 expression, thereby promoting NSC proliferation and neuronal differentiation. More broadly, our study provides new insights into the development of versatile therapeutic targets that may facilitate both the prevention and treatment of neurodegenerative diseases associated with cognitive impairment.

### Limitations of the study

There are still several issues to be further explored. First, there is no direct evidence currently indicating that changes in miR-544-3p would affect cognitive function. Therefore, it remains unclear whether alterations in miR-544-3p *in vivo* would have an impact on cognitive function. Second, whether the continuous upregulation of the glucocorticoid receptor encoded by Nr3c1 would lead to increased stress sensitivity or metabolic changes also requires further exploration. Third, whether miR-544-3p/Nr3c1 would change with the overexpression of circCdyl in animal models. Fourthly, whether circCdyl would induce NSC differentiation into specific neuronal subtypes and integrate them into neuronal circuits.

## Resource availability

### Lead contact

Further information and requests for resources and reagents should be directed to and will be fulfilled by the lead contact, Xinhua Zhang (zhangxinhua@ntu.edu.cn).

### Materials availability

Materials generated in this study, including cell lines and plasmids, will be available upon request of the lead contact.

### Data and code availability

• The RNA-seq raw data are deposited on the Gene Expression Omnibus (GEO) database (GEO accession number: GSE311822).
• This article does not report original code.
• Any additional information required to reanalyze the data reported in this article is available from the lead contact upon request.

## STAR★Methods

### Key resources table

**Table:** 

REAGENT or RESOURCE	SOURCE	IDENTIFIER
**Antibodies**		

polyclonal anti-BrdU	Abcam	ab152095
Alexa Fluor® 647 Mouse anti-GFAP	BD Biosciences	550524
Monoclonal anti-Tuj1	Abcam	ab78078
monoclonal anti- MAP2	Abcam	ab5392
polyclonal anti-Nr3C1	Abcam	ab183127
monoclonal anti-β-actin	Proteintech	HRP-60008
polyclonal anti-Ki67	Abcam	ab15580
monoclonal anti-Nestin	Milipore	MAB353
monoclonal anti-Tuj1	Milipore	MAB1637
monoclonal anti-NeuN	Abcam	ab177487

**Bacterial and virus strains**		

circCdyl overexpression lentivirus	GeneChem	N/A

**Chemicals, peptides, and recombinant proteins**		

B27 supplement	Gibco	12587010
basic fibroblast growth factor (bFGF)	Sigma	GF003AF
epidermal growth factor (EGF)	Sigma	E1235
Ham F-12 nutrient mixture (1:1, DMEM/F-12, Gibco)	Gibco	8122199
fetal bovine serum (Gibco)	Gibco	A5670701
BrdU (5-bromo-2′-deoxyuridine), Thymidine analog	Abcam	ab142567
TRIzol Reagent	Invitrogen	15596026
AceQ qPCR SYBR Green Master Mix (Vazyme)	Vazyme	Q141
actinomycin D	MCE	Y-17559
Hoechst 33342	Sigma	14533
RNase R	Beyotime	R7092M
PI/RNase Staining Buffer	BD Biosciences	550825
M-PER^TM^ Mammalian Protein Extraction Reagent	Thermo	78501
PAGE Gel Fast Preparation Kit	Epizyme	PG112/PG111
ECL chemiluminescence reagent	Millipore	WBKLS0500
Lipofectamine 3000	Invitrogen	L3000075

**Critical commercial assays**		

HiScript II 1st Strand cDNA Synthesis Kit	Vazyme	R211
miRNA First-Strand cDNA kit	TIANGEN	KR211
miRcute Plus miRNA qPCR Kit	TIANGEN	FP411
Cell Counting Kit 8 (CCK8)	Beyotime	C0038
Fluorescent *in situ* Hybridization kit	Genepharma	N/A
Dual Luciferase Reporter Assay Kit	Promega	E1910

**Deposited data**		

RNA sequencing data	This paper	GSE311822

**Experimental models: Cell lines**		

293T	ATCC	CRL-3216

**Experimental models: Organisms/strains**		

Sprague-Dawley (SD) rats	Experimental Animal Center of Nantong University	N/A

**Oligonucleotides**		

Primers for qPCR, see Table S1	Sangon Biotech	N/A
Cy3-labeled circCdyl probes	Genepharma	N/A
FAM-labeled rno-miR-544-3p probes	Genepharma	N/A
Si-Ctrl or OE-Ctrl	RiboBio	N/A
Si-Nr3c1 or OE-Nr3c1	RiboBio	N/A
MicroRNA mimic or inhibitor Negative Control	RiboBio	N/A
miR-544-3p mimic or miR-544-3p inhibitor	RiboBio	N/A

**Software and algorithms**		

Graphpad Prism9.0	GraphPad Software	https://www.graphpad.com
ImageJ	ImageJ software	https://imagej.net/software/fiji/
EndNote X9	EndNote X9 Software	https://endnote.com/downloads
Adobe Photoshop	Adobe	https://www.adobe.com/es/

### Experimental model and study participant details

#### Rat and experimental protocols

Pregnant 15d Sprague-Dawley (SD) rats, and adult SD rats (220–250g) were obtained from the Experimental Animal Center of Nantong University, China (Certificate No: SYXK (SU) 2019-0001). Both male and female rats were used for experiments. All animal experiments were performed in accordance with the guidelines of the National Institutes of Health, and were approved by the Animal Use and Care Committee of Nantong University.

Subjects (n=60) were adult male SD rats. After anesthetized with a mixture of enflurane: oxygen: nitrous oxide (1:33:66), the rats were transferred to the stereotaxic apparatus rats, and FF transection was performed with a wire knife on the dorsal side of hippocampal CA1 layer, at coordinates of bregma: anteroposterior (AP) = 1.4, mediolateral (ML) = 1.0 and AP = 1.4, ML = 4.0 (right); AP = 1.4, ML = −1.0 and AP = 1.4, ML = −4.0 (left), and depth 5.4 to 5.6 mm.

At day 14 after FF transection, lentivirus in a total volume of 5 μL were microinjected into hippocampal DG at two points: 3.6 mm to bregma, 1.39 mm to the right or left of the midline, and 3.9 mm in depth. The speed of the injection was 0.5 μL/min. After the injection, the needle was kept in the position for an additional 10min, and then retrieved slowly out of the brain.

#### Cell culture

NSCs were isolated from the embryonic day 15 (E15) SD rat hippocampus as described previously.^18^ After dissociated and resuspension, cells were cultured in proliferation medium containing 2% B27 supplement (Gibco), 20 ng/ml basic fibroblast growth factor (bFGF; Sigma), 20 ng/ml epidermal growth factor (EGF; Sigma), Ham F-12 nutrient mixture (1:1, DMEM/F-12, Gibco) and passaged every 5-7 days. For NSC differentiation, NSCs were plated at plates in DMEM/F12 (1:1) medium containing 2% B27 and 2% fetal bovine serum (Gibco).

Human embryonic kidney cells (HEK-293T) were maintained in our laboratory, which were purchased from ATCC. HEK-293T cells were cultured in DMEM (Gibco, China) supplemented with 10% FBS. All cells were cultured in a humidified incubator at 37°C with 5% CO_2_.

### Method details

#### Lentiviral transduction and transfection

To construct overexpression vector, the linear sequence of circCdyl was inserted into the (poly A-MCS-UBI) RV-SV40-EGFP-IRES-puromycin vector, whereas the mock vector with no target gene sequence was used as a control (GeneChem). NSCs were transduced with control and circCdyl overexpression lentivirus with multiplicity of infection of 10.

The control, Nr3c1 siRNA, pcDNA Nr3c1, miR-544-3p mimic (50 nM), and miR-544-3p inhibitor (100 nM) were transfected into the cells using Lipofectamine 3000 (Invitrogen) according to the manufacturer’s instructions.

#### BrdU incorporation assay

*In vivo* assays for BrdU incorporation used the BrdU Staining Kit from Abcam according to the manufacture’s protocol. In brief, rats were intraperitoneal injected with BrdU at 50mg/kg daily for 7 consecutive days. Then, fix brain tissue samples were permeabilized with 0.01% Triton X-100, blocked in 2% bovine serum albumin (BSA), and stained using polyclonal anti-BrdU antibody (1:1000; Abcam) overnight at 4°C, and Alexa Fluor568-conjugated goat anti-mouse IgG, were added and incubated for 2 h, then dyed by Hoechst 33342 (1:1000; Sigma) and observed by a fluorescence microscope (Zeiss).

#### Total RNA extraction and qRT-PCR

Total RNA was extracted by TRIzol Reagent (Invitrogen), reverse-transcribed according to the cDNA first strand kit (Vazyme), and miRNA First-Strand cDNA kit (TIANGEN) protocols. qRT-PCR was performed with AceQ qPCR SYBR Green Master Mix (Vazyme) and miRcute Plus miRNA qPCR Kit (TIANGEN) using StepOnePlus^TM^ Real-Time PCR System (Thermo Fisher Scientific). mRNA primers (Sangon Biotech) were listed in Table S1. circRNA and miRNAs primers were designed and synthesized by RiboBio. The intellectual property rights of the primer sequence belong to Ribo biology, which were asked to be classified. The 2^−ΔΔ^Ct was used to quantify the expression of target genes.

#### Sanger sequencing, actinomycin D, and RNase R treatment

The circCdyl sequence was obtained using divergent primers, and Sanger sequencing was performed by Geneseed Biotech Co.

NSCs were treated with 2 mg/mL actinomycin D (MCE) to block transcription. Then the cells were harvested, and total RNA was extracted. The stability of circCdyl and Cdyl mRNA were were assessed by qRT-PCR.

Total RNA (5 μg) was incubated with 3 U/μg RNase R (Beyotime) for 20 min at 37°C. Then, followed by qRT-PCR analysis to detect circCdyl expression levels.

#### Flow cytometry

For cell cycle detection, NSCs were dissociated with 0.25% trypsin, and then fixed in ice-cold 75% ethyl alcohol overnight at 4°C. Then, 400 μL of PI/RNase Staining Buffer (BD Biosciences) was added and stained for 30 min, followed by detection with FACS Calibur (BD Biosciences).

Cells were trypsinized and collected, and fixed in 1× Fix/Perm Buffer at 4°C for 40 min. After washing with 1× Perm/Wash Buffer, cell samples were mixed with 100 μl of 1× Perm/Wash Buffer and incubated with anti-GFAP antibody (5 μl/test) (BD Biosciences) at 4°C for 2 h, followed by detection with FACS Calibur (BD Biosciences).

#### CCK8 assay

Cells were cultured in 96-well plates with 2×10^4^ cells/well. The Cell Counting Kit 8 (CCK8, C0038, Beyotime, China) was used to evaluate cell proliferation in accordance with the manufacturer’s instructions. After 2 h of incubation at 37°C, the plates were evaluated at 450nm using Synergy2 multi-function microplate reader (BioTek, USA).

#### Western blot

Cells were collected and extracted by M-PER^TM^ Mammalian Protein Extraction Reagent (Thermo). Protein sample were separated by PAGE Gel Fast Preparation Kit (Epizyme) and transferred to PVDF (Merck, Ireland). PVDF was incubated in 5% skim milk powder at room temperature for 2h. Monoclonal anti-Tuj1 (1:1000; ab78078, Abcam), monoclonal anti- MAP2 (1:1000; ab5392, Abcam), polyclonal anti-Nr3C1 (1:1000; ab183127, Abcam), monoclonal anti-β-actin (1:5000; HRP-60008, Proteintech) antibodies were used, and proteins were visualized by ECL chemiluminescence reagent (Millipore).

#### Immunofluorescence

After treatment as indicated, cells were fixed with 4% PFA. Then, cells or fix brain tissue samples were permeabilized with 0.01% Triton X-100, and blocked in 2% BSA. Cell climbing pieces or fix brain tissue samples were incubated with polyclonal anti-Ki67 (1:200; ab15580, Abcam), monoclonal anti-Nestin (1:200; MAB353, Milipore), monoclonal anti-Tuj1 (1:1000; MAB1637, Milipore), polyclonal anti-Nr3c1 (1:1000; Ab183127, Abcam), monoclonal anti-NeuN (1:1000; ab177487, Abcam), antibodies overnight at 4°C, and Alexa Fluor568-conjugated goat anti-mouse, or goat anti-rabbit IgG, Alexa Fluor488 conjugated goat anti-mouse, or goat anti-rabbit IgG (1:1000; Invitrogen) were added and incubated for 2 h, then dyed by Hoechst 33342 (Sigma) and observed by a fluorescence microscope (Zeiss).

#### Fluorescent *in situ* hybridization (FISH)

Cy3-labeled circCdyl (ACTTTCCACCTTTTCCATTAACAG-3′) and FAM-labeled rno-miR-544-3p probes (5′-AGCTTGCTAAAAATGCAGAAT-3′) (Genepharma) were used to observe the location of circCdyl and rno-miR-544-3p in NSCs. Follow the instructions of the Fluorescent *in situ* Hybridization kit (Genepharma) and the images were photographed with a confocal microscope (Zeiss).

#### Dual luciferase reporter assay

The sequences of circCdyl or Nr3c1 3′UTR containing the wild-type (WT) or mutant (Mut) binding site of miR-544-3p were devised and synthesized by GenePharma (Shanghai, China). 293T cells were co-transfected with the corresponding plasmids and miR-544-3p mimic/miR-NC with Lipofectamine 3000 (Invitrogen). The experimental steps follow the manufacturer’s protocols of Dual Luciferase Reporter Assay Kit (Promega). The ratio of firefly to Renilla luciferase activity was subsequently determined.

#### Morris water maze

The Morris water maze test was initiated on day 35 post-treatment and continued for five consecutive days. SD rats from five experimental groups (n = 10 per group) were tested in a circular pool divided into four equal quadrants: northeast (NE), northwest (NW), southeast (SE), and southwest (SW). Escape latency, path length, and swimming speed were recorded during the acquisition phase. On day 5, a probe trial was conducted in which the platform was removed, and the number of crossings over the original platform location, as well as the time spent and path length traveled in the target quadrant, were measured.

#### Passive and active avoidance task

Each rat was allowed a 10 min acclimatization period with free access to either the light or dark compartment of the avoidance training box. During the conditioning phase, rats were placed into the illuminated compartment, and the guillotine door was opened after 30 s. Upon entering the dark compartment, the door was closed, and a foot shock (5 s, 1.5 mA) was delivered through the grid floor. After 20 s, the rat was removed from the dark compartment and returned to its home cage. To assess short-term memory, testing was conducted 24 h after training. Each animal was placed again into the light compartment, and 30 s later, the door was raised. The latency to enter the dark compartment—defined as step-through latency—was recorded. The trial ended when the animal entered the dark compartment or remained in the light compartment for 300 s, whichever occurred first. No electric shock was administered during this retention test.

Each rat was placed in the left compartment, and facing the end wall. After 20 s, a trial started with conditioned stimulus (CS, light of 7 W and sound of 2400 Hz at 40 dB, presented simultaneously) onset and, 5 s later, followed by a 1.6 mA intensity foot shock (unconditioned stimulus, US). If the rat moved to the opposite compartment during the CS–US interval, the trial was immediately terminated and scored as an avoidance response. If the rat crossed after the onset of the US, the behavior was recorded as an escape response.

### Quantification and statistical analysis

Statistical analysis was performed using GraphPad Prism 9.0 software. Each experimental group comprised at least three independent replicates. All data are presented as mean ± standard deviation (SD). Group comparisons between two conditions were conducted using the paired t-test, and multiple-group comparisons were performed using one-way ANOVA. Statistical significance was defined as ∗*P* < 0.05, ∗∗*P* < 0.01, ∗∗∗*P* < 0.001.

## Acknowledgments

The present study was supported by the Graduate Scientific Research Innovation Program of Jiangsu Province(grant no. KYCX19 2066), the 10.13039/501100001809National Natural Science Foundation of China (grant no. 31171038), the 10.13039/501100004608Jiangsu Natural Science Foundation (grant no. BK2011385), the Jiangsu “333” Program funding (grant no. BRA2016450), the Application Research Project of Nantong City (grant no. MS12017015-3), The Training Program of Innovation and Entrepreneurship for Graduates of 10.13039/501100005054Nantong University of China (grant no. 265), a Project Funded by the 10.13039/501100012246Priority Academic Program Development of Jiangsu Higher Education Institutions (10.13039/501100012246PAPD) of Jiangsu Higher Education Institutions (grant no. 03081023), 10.13039/501100018557Nantong Science and Technology Project (grant no. JC2021056), and the Innovation and Entrepreneurship Training Program for College Students (grant no. 202310304046Z).

## Author contributions

Conceptualization, W.L., Y.L., J.W., and X.Z.; methodology, Z.Z., J.Z., Y.Z., Y.G., T.Y., X.C., W.C., and M.X.; formal analysis, W.L., Y.L., and J.W.; investigation, W.L., Y.L., and J.W.; resources, Z.Z., J.Z., Y.Z., Y.G., T.Y., X.C., W.C., and M.X.; writing – original draft, W.L., Y.L., and J.W., data Curation, Z.Z., J.Z., Y.Z., Y.G., T.Y., X.C., W.C., and M.X.; project administration, W.L. and X.Z.; funding acquisition, W.L. and X.Z.; writing-review & editing, W.L. and X.Z.

## Declaration of interests

The authors declare no competing interests.

## References

[1] Sekeres M.J., Bradley-Garcia M., Martinez-Canabal A., Winocur G. (2021). Chemotherapy-Induced Cognitive Impairment and Hippocampal Neurogenesis: A Review of Physiological Mechanisms and Interventions. Int. J. Mol. Sci..

[2] Obernier K., Alvarez-Buylla A. (2019). Neural stem cells: origin, heterogeneity and regulation in the adult mammalian brain. Development.

[3] Tobin M.K., Musaraca K., Disouky A., Shetti A., Bheri A., Honer W.G., Kim N., Dawe R.J., Bennett D.A., Arfanakis K., Lazarov O. (2019). Human Hippocampal Neurogenesis Persists in Aged Adults and Alzheimer's Disease Patients. Cell Stem Cell.

[4] Moreno-Jimenez E.P., Flor-Garcia M., Terreros-Roncal J., Rabano A., Cafini F., Pallas-Bazarra N., Avila J., Llorens-Martin M. (2019). Adult hippocampal neurogenesis is abundant in neurologically healthy subjects and drops sharply in patients with Alzheimer's disease. Nat Med.

[5] Soto J., Ding X., Wang A., Li S. (2021). Neural crest-like stem cells for tissue regeneration. Stem Cells Transl. Med..

[6] Bonaventura G., Munafo A., Bellanca C.M., La Cognata V., Iemmolo R., Attaguile G.A., Di Mauro R., Di Benedetto G., Cantarella G., Barcellona M.L. (2021). Stem Cells: Innovative Therapeutic Options for Neurodegenerative Diseases?. Cells.

[7] Chen L., Wang C., Sun H., Wang J., Liang Y., Wang Y., Wong G. (2021). The bioinformatics toolbox for circRNA discovery and analysis. Brief. Bioinform..

[8] Chen Z., Song M., Wang T., Gao J., Lin F., Dai H., Zhang C. (2022). Role of circRNA in E3 Modification under Human Disease. Biomolecules.

[9] Mahmoudi E., Cairns M.J. (2019). Circular RNAs are temporospatially regulated throughout development and ageing in the rat. Sci. Rep..

[10] Sekar S., Liang W.S. (2019). Circular RNA expression and function in the brain. Noncoding. RNA Res..

[11] Mehta S.L., Dempsey R.J., Vemuganti R. (2020). Role of circular RNAs in brain development and CNS diseases. Prog. Neurobiol..

[12] Wu F., Han B., Wu S., Yang L., Leng S., Li M., Liao J., Wang G., Ye Q., Zhang Y. (2019). Circular RNA TLK1 Aggravates Neuronal Injury and Neurological Deficits after Ischemic Stroke via miR-335-3p/TIPARP. J. Neurosci..

[13] Qi X., Chen X., Zhao Y., Chen J., Niu B., Shen B. (2022). Prognostic Roles of ceRNA Network-Based Signatures in Gastrointestinal Cancers. Front. Oncol..

[14] He L., Zhang F., Zhu Y., Lu M. (2022). A crosstalk between circular RNA, microRNA, and messenger RNA in the development of various brain cognitive disorders. Front. Mol. Neurosci..

[15] Feng Z., Zhang L., Wang S., Hong Q. (2020). Circular RNA circDLGAP4 exerts neuroprotective effects via modulating miR-134-5p/CREB pathway in Parkinson's disease. Biochem. Biophys. Res. Commun..

[16] Li W., Shan B., Cheng X., He H., Qin J., Zhao H., Tian M., Zhang X., Jin G. (2022). circRNA Acbd6 promotes neural stem cell differentiation into cholinergic neurons via the miR-320-5p-Osbpl2 axis. J. Biol. Chem..

[17] Cheng X., Li W., Zhao R., Li H., Qin J., Tian M., Zhang X., Jin G. (2021). The role of hippocampal niche exosomes in rat hippocampal neurogenesis after fimbria-fornix transection. J. Biol. Chem..

[18] Li W., Wang S.S., Shan B.Q., Qin J.B., Zhao H.Y., Tian M.L., He H., Cheng X., Zhang X.H., Jin G.H. (2022). miR-103-3p targets Ndel1 to regulate neural stem cell proliferation and differentiation. Neural Regen. Res..

[19] Mannino G., Russo C., Maugeri G., Musumeci G., Vicario N., Tibullo D., Giuffrida R., Parenti R., Lo Furno D. (2022). Adult stem cell niches for tissue homeostasis. J. Cell. Physiol..

[20] Morante-Redolat J.M., Porlan E. (2019). Neural Stem Cell Regulation by Adhesion Molecules Within the Subependymal Niche. Front. Cell Dev. Biol..

[21] Matsubara S., Matsuda T., Nakashima K. (2021). Regulation of Adult Mammalian Neural Stem Cells and Neurogenesis by Cell Extrinsic and Intrinsic Factors. Cells.

[22] Weerasinghe-Mudiyanselage P.D.E., Ang M.J., Kang S., Kim J.S., Moon C. (2022). Structural Plasticity of the Hippocampus in Neurodegenerative Diseases. Int. J. Mol. Sci..

[23] Zou L., Jin G., Zhang X., Qin J., Zhu H., Tian M., Tan X. (2010). Proliferation, migration, and neuronal differentiation of the endogenous neural progenitors in hippocampus after fimbria fornix transection. Int. J. Neurosci..

[24] Patop I.L., Wüst S., Kadener S. (2019). Past, present, and future of circRNAs. EMBO J..

[25] Tan Z., Li W., Cheng X., Zhu Q., Zhang X. (2022). Non-Coding RNAs in the Regulation of Hippocampal Neurogenesis and Potential Treatment Targets for Related Disorders. Biomolecules.

[26] D'Anca M., Buccellato F.R., Fenoglio C., Galimberti D. (2022). Circular RNAs: Emblematic Players of Neurogenesis and Neurodegeneration. Int. J. Mol. Sci..

[27] Rybak-Wolf A., Stottmeister C., Glažar P., Jens M., Pino N., Giusti S., Hanan M., Behm M., Bartok O., Ashwal-Fluss R. (2015). Circular RNAs in the Mammalian Brain Are Highly Abundant, Conserved, and Dynamically Expressed. Mol. Cell.

[28] You X., Vlatkovic I., Babic A., Will T., Epstein I., Tushev G., Akbalik G., Wang M., Glock C., Quedenau C. (2015). Neural circular RNAs are derived from synaptic genes and regulated by development and plasticity. Nat. Neurosci..

[29] Li Y., Zheng Q., Bao C., Li S., Guo W., Zhao J., Chen D., Gu J., He X., Huang S. (2015). Circular RNA is enriched and stable in exosomes: a promising biomarker for cancer diagnosis. Cell Res..

[30] Wang Y., Liu J., Ma J., Sun T., Zhou Q., Wang W., Wang G., Wu P., Wang H., Jiang L. (2019). Exosomal circRNAs: biogenesis, effect and application in human diseases. Mol. Cancer.

[31] Tian L., Cao J., Jiao H., Zhang J., Ren X., Liu X., Liu M., Sun Y. (2019). CircRASSF2 promotes laryngeal squamous cell carcinoma progression by regulating the miR-302b-3p/IGF-1R axis. Clin. Sci..

[32] Zhang X., Wang S., Wang H., Cao J., Huang X., Chen Z., Xu P., Sun G., Xu J., Lv J., Xu Z. (2019). Circular RNA circNRIP1 acts as a microRNA-149-5p sponge to promote gastric cancer progression via the AKT1/mTOR pathway. Mol. Cancer.

[33] Zhao R.T., Zhou J., Dong X.L., Bi C.W., Jiang R.C., Dong J.F., Tian Y., Yuan H.J., Zhang J.N. (2018). Circular Ribonucleic Acid Expression Alteration in Exosomes from the Brain Extracellular Space after Traumatic Brain Injury in Mice. J. Neurotrauma.

[34] Zhang Y., Yu F., Bao S., Sun J. (2019). Systematic Characterization of Circular RNA-Associated CeRNA Network Identified Novel circRNA Biomarkers in Alzheimer's Disease. Front. Bioeng. Biotechnol..

[35] Yang Y., Wang Y., Qu H., Wang D., Ge J., Wu P., Yan Q., Chen P., Xiang B., Zhou M. (2025). CircCDYL promotes glycolysis to drive the progression of nasopharyngeal carcinoma. J. Adv. Res..

[36] Wei Y., Chen X., Liang C., Ling Y., Yang X., Ye X., Zhang H., Yang P., Cui X., Ren Y. (2020). A Noncoding Regulatory RNAs Network Driven by Circ-CDYL Acts Specifically in the Early Stages Hepatocellular Carcinoma. Hepatology.

[37] Li M., Ding W., Fang X., Wang Y., Wang P., Ye L., Miao S., Song L., Ao X., Li Q., Wang J. (2025). Novel Truncated Peptide Derived From circCDYL Exacerbates Cardiac Hypertrophy. Circ. Res..

[38] Qin R., Cao S., Lyu T., Qi C., Zhang W., Wang Y. (2017). CDYL Deficiency Disrupts Neuronal Migration and Increases Susceptibility to Epilepsy. Cell Rep..

[39] Wadji D.L., Tandon T., Ketcha Wanda G.J.M., Wicky C., Dentz A., Hasler G., Morina N., Martin-Soelch C. (2021). Child maltreatment and NR3C1 exon 1(F) methylation, link with deregulated hypothalamus-pituitary-adrenal axis and psychopathology: A systematic review. Child Abuse Negl..

