# Supplementary material for: CircRNA Cdyl promotes the proliferation and differentiation of neural stem cells via regulating miR-544-3p/Nr3c1 axis
Source: iScience. 2026 Jan 15;29(2):114716. doi: 10.1016/j.isci.2026.114716 (PMC12886517; doi:10.1016/j.isci.2026.114716)
Supplement: Document S1. Figures S1–S3 and Table S1 [file mmc1.pdf]

iScience, Volume *29*

Supplemental information

CircRNA Cdyl promotes the proliferation and differentiation of neural stem cells via regulating miR-544-3p/Nr3c1 axis

Wen Li, Yujian Lin, Jingwen Wang, Zuotian Zhang, Jingjing Zhang, Yuzheng Zhang, Yuxuan Ge, Tiankun Yao, Xiang Cheng, Weiwei Chen, Min Xu, and Xinhua Zhang

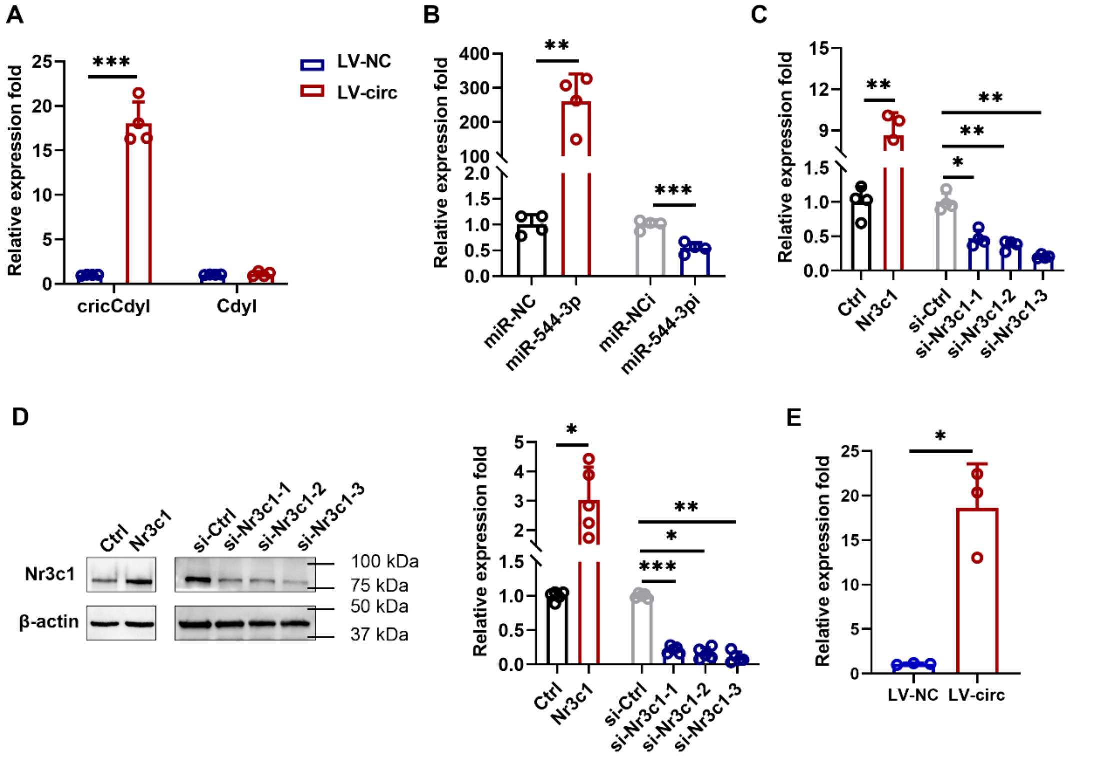

Figure S1. Transfection and infection efficiency of circCdyl, Nr3c1, and miR-544-3p (A) qRT-PCR was conducted to determine the abundances of circCdyl in NSCs infected with circCdyl overexpression of lentivirus. (LV-NC) NSCs infected with control lentivirus;(LV-circ) NSCs infected with circCdyl overexpression of lentivirus. (B) qRT-PCR was conducted to determine the abundances of miR-544-3p in NSCs transfected with miR-544-3p. (miR-NC) NSCs transfected with control miRNA mimic; (miR-544-3p) NSCs transfected with miR-544-3p mimic; (miR-NCi) NSCs transfected with control miRNA inhibitor; (miR-544-3pi) NSCs transfected with miR-544-3p inhibitor. (C, D) qRT-PCR and Western blot were conducted to determine the abundances of Nr3c1 in NSCs transfected with Nr3c1. (Ctrl) NSCs transfected with control pcDNA; (Nr3c1) NSCs transfected with pcDNA Nr3c1; (si-Ctrl) NSCs transfected with control siRNA; (si-Nr3c1) NSCs transfected with Nr3c1 siRNA. (E) qRT-PCR was conducted to determine the abundances of circCdyl in hippocampus infected with circCdyl overexpression of lentivirus. (LV-NC) hippocampus infected with control lentivirus;(LV-circ) hippocampus infected with circCdyl overexpression of lentivirus. n = 3–5 in each group. All data are presented as mean ± SD and analyzed by paired Student's t test. *$P < 0.05$, **$P < 0.01$, ***$P < 0.001$, values significantly different from corresponding control group.

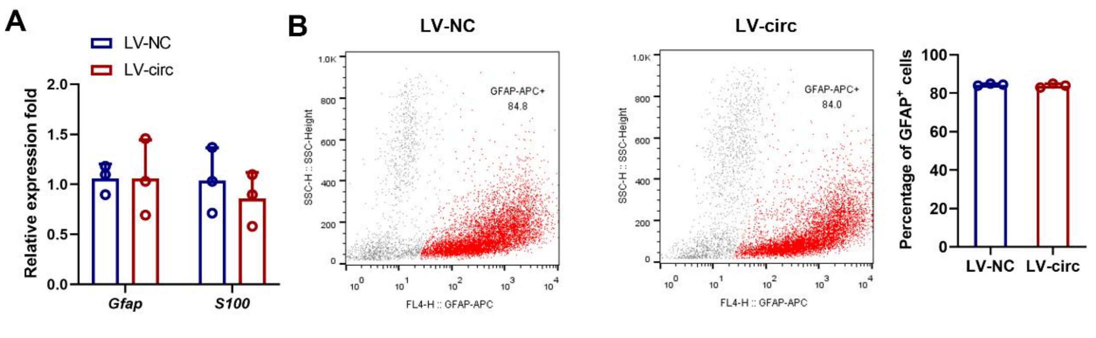

Figure S2. The influence of CircCdyl on the differentiation of NSCs into astrocytes
(A, B) qRT-PCR and flow cytometry was conducted to determine the expression of astrocytic marker was measured by qRT-PCR. (LV-NC) NSCs infected with control lentivirus;(LV-circ) NSCs infected with circCdyl overexpression of lentivirus. n = 3 in each group. All data are presented as mean ± SD and analyzed by paired Student’s t test.

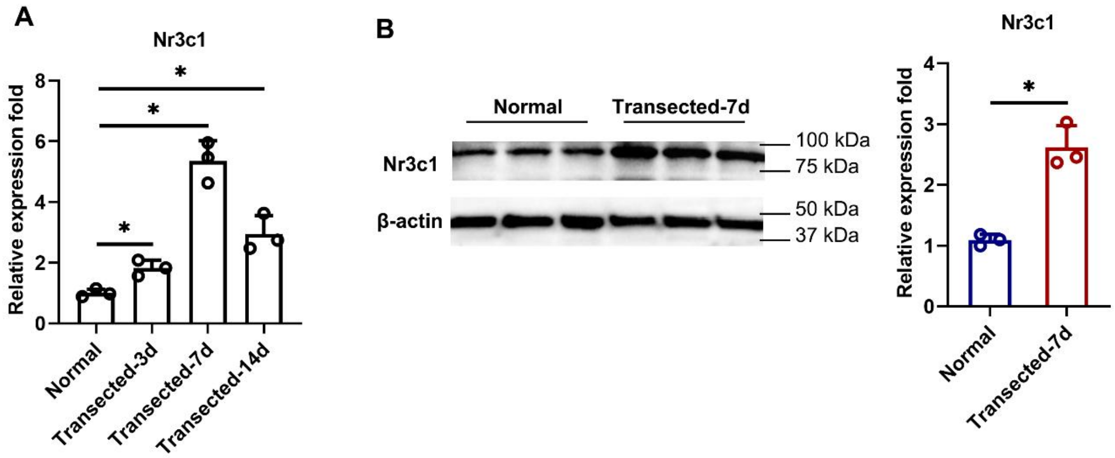

Figure S3. The expression of Nr3c1 in the hippocampus

(A, B) After FF transection, the expression of Nr3c1 in the hippocampus was also detected by qRT-PCR and western blot. n = 3 in each group. All data are presented as mean ± SD and analyzed by paired Student's t test. **P* < 0.05, values significantly different from corresponding control group.

Supplementary Table

Table S1. Primers used for qRT-PCR, related to STAR Methods

Name	Sequence(5'-3')
Bloc1s5	Forward: CCAACCATGTAGCCTCTCCTTCAAG<br>Reverse: CCTCCTCTTCCTCAGACGACCAC
Ccnd1	Forward: CCCTCGGTGTCCTACTTCAA<br>Reverse: GTGTTCAATGAAATCGTGCG
circCDYL	Forward: AGCCGGTCGGAGCTTTATTG<br>Reverse: TCCTTTCAACCTTTCCCGTTAAC
Commd9	Forward: ACCCTAGTCTGTGTGGAGAGAAACC<br>Reverse: GAATGCGACCCAGCCCATCTAAC
Cops2	Forward: TGAGGAGGACTACGACCTGGAATAC<br>Reverse: TTAGTGCTGCTTTCGGGTCATCTTC
Gapdh	Forward: CGGGAAGGAAATGAATGGGCAG<br>Reverse: TAGCCTCGCTCCACCTGACTT
Gfap	Forward: CTCAAGAGGAACACTGTGGTAAAGA<br>Reverse: CTGAACGAGCCGTGGGCATAAAA
Gpbp1	Forward: TGCCTCGGTGATTTCTCAACAGATC<br>Reverse: TGTCTTCCTGCCAGCCCATCTC
Hsdl2	Forward: ACTCACAGGTCGCTGCTGTT<br>Reverse: ACTGTGCATCCCGCTAGCTT
Ki67	Forward: ACCATCATTGACCGCTCCTTT<br>Reverse: AGGCCCTTGGCATACACAAA
Map2	Forward: CTTGATTCTATTGCCCTTGGGTTTA<br>Reverse: CATCCATCGTTCCGCTAGTGTTG
Mcm2	Forward: GGTACTGCTATGGCGGAATCATC<br>Reverse: AAATGGTGGAAGGTCACGGC
Neun	Forward: CATGGGTCACAGGGCACCTATTC<br>Reverse: GGTGGAGAAGGAGGCAGATTAGCTG
Mocs2	Forward: AGTTGCCATCATCCCACCAATCAG<br>Reverse: AGGTTTCTCCAGCACATCATCCAC
Nr3c1	Forward: CACATCTCACCCGCACCGATTG<br>Reverse: TTGGACAAACACGGATGCCTGAC
Pcna	Forward: AAAGATGCCGTCGGGTGAAT<br>Reverse: TGGTTACCGCCTCCTCTTCT
Rbm25	Forward: TCCGCTCCATCTGTGTCCTCTG<br>Reverse: CAGGCTGCTGTTGATCTGGTGAG
Syn1	Forward: CCTCATTCGTGCTGCCTGTGGTA<br>Reverse: CTGGGCCTTTGCTTGTTTATTTT
Tuj1	Forward: CGCCATGTTCAGACGCAAG<br>Reverse: CTCGGACACCAGGTCGTTCA
